# Direct Analysis in Real Time-Mass Spectrometry Measurement of Antifungals in Human Serum: A Proof-of-Concept Study

**DOI:** 10.21627/x6vfj859

**Published:** 2026-07-10

**Authors:** Julie A. Ray, Stephen D. Merrigan, Preejith Vachali, Terry L. Bates, Zahuindanda Aventura, Samuel Putnam, Gwendolyn McMillin, Mark A. Fisher, Kamisha L. Johnson-Davis

**Affiliations:** ARUP Institute for Clinical and Experimental Pathology, Salt Lake City, UT 84108, United States; ARUP Institute for Clinical and Experimental Pathology, Salt Lake City, UT 84108, United States; ARUP Institute for Clinical and Experimental Pathology, Salt Lake City, UT 84108, United States; Bruker Scientific LLC, Billerica, MA 01821, United States; Bruker Daltonics GmbH, Bremen, Germany; Bruker Scientific LLC, Billerica, MA 01821, United States; ARUP Institute for Clinical and Experimental Pathology, Salt Lake City, UT 84108, United States; Department of Pathology, University of Utah Health, Salt Lake City, UT 84108, United States; ARUP Institute for Clinical and Experimental Pathology, Salt Lake City, UT 84108, United States; Department of Pathology, University of Utah Health, Salt Lake City, UT 84108, United States; ARUP Institute for Clinical and Experimental Pathology, Salt Lake City, UT 84108, United States; Department of Pathology, University of Utah Health, Salt Lake City, UT 84108, United States

**Keywords:** Direct analysis in real time, DART-mass spectrometry, antifungals, LC-MS, protein precipitation, liquid-liquid extraction

## Abstract

**INTRODUCTION:**

Direct analysis in real time (DART) is a soft ionization technique that uses a heated gas such as helium, argon, or nitrogen to generate a cascade of ionized atmospheric molecules that subsequently ionize analytes in biological samples.

**OBJECTIVES:**

In this proof-of-concept study evaluating selected antifungals in human serum, 5-fluorocytosine (5FC), itraconazole (ITR), hydroxyitraconazole (OH-ITR), isavuconazole (ISV), ketoconazole (KETO), fluconazole (FLU), and posaconazole (POSA) were assessed as DART candidates using a Bruker EVOQ® DART-TQ+.

**METHODS:**

Human serum samples were extracted using protein precipitation or liquid–liquid extraction. Quantification was performed by DART tandem mass spectrometry (DART-MS/MS) with internal standards. Precision and linearity across individual analytical measurement ranges (AMRs) were evaluated with rapid sample acquisition (0.24 min linear scans), and the results were compared with those obtained using validated LC-MS/MS methods.

**RESULTS:**

Linearity and precision were acceptable for all evaluated compounds. Good agreement with LC-MS/MS was observed for 5FC, ITR, OH-ITR, and FLU, with slopes ranging from 0.875 to 1.112 and correlation coefficients (*r*) ≥ 0.949. POSA extraction by protein precipitation resulted in high bias; however, liquid–liquid extraction or the use of an alternative ion transition reduced bias to <7%. DART replicates demonstrated coefficients of variation <15%. All samples met ion-ratio tolerance criteria of ±50%.

**CONCLUSION:**

This proof-of-concept study demonstrates the feasibility of quantitative antifungal measurement in human serum using DART-MS/MS with simple sample preparation. A moderate linear association with bias for POSA was likely attributable to matrix effects. More selective sample preparation may improve DART accuracy. Overall, DART-MS/MS shows promise as a rapid, high-throughput alternative to conventional LC-MS/MS.

## Non-Specialist Summary

Researchers tested a fast mass spectrometry technique (direct analysis in real time tandem mass spectrometry, DART-MS/MS) to measure antifungal drugs in human blood serum. Using simple extraction steps, they analyzed seven common medications and compared the results with those obtained using a standard liquid chromatography tandem mass spectrometry (LC-MS/MS) lab method. For most drugs, accuracy, precision, and linearity were good and matched those of the reference method. Posaconazole showed some bias, likely from sample matrix effects. Overall, DART-MS/MS appears promising for quick, high-throughput drug level monitoring.

## 1. Introduction

Direct analysis in real time (DART), introduced in 2005 [1], is a plasma-based ambient ionization approach that enables soft ionization of analytes at atmospheric pressure. Long-lived metastable species are generated within the DART source, most commonly using helium, and released into the ambient environment, where they initiate a reaction cascade that can include Penning ionization [2] and other gas-phase processes. This cascade ultimately yields charged reagent ions that facilitate the ionization of target compounds in either positive or negative mode. Ambient ionization by DART allows for samples to be ionized without being introduced into a vacuum. It is rapid and provides real-time results, where the analysis time is measured in seconds. Although DART offers advantages for certain analyses, other rapid, ambient ionization techniques with comparable throughput potential also exist. Atmospheric-pressure matrix-assisted laser desorption/ionization (AP-MALDI) is limited by time-consuming sample preparation steps, particularly matrix co-crystallization, which can introduce substantial sample-to-sample variability that might not be corrected by internal standard normalization [3]. Desorption electrospray ionization (DESI) has also been limited by reproducibility challenges, particularly when applied to dried biological extracts, where surface heterogeneity, droplet–substrate interactions, and spray geometry can introduce substantial variability in signal intensity [4, 5].

Although conventionally considered a qualitative analysis tool, DART-MS has recently been demonstrated to be suitable as a quantitative tool for appropriate analyses. DART combined with tandem mass spectrometry (DART-MS/MS) can be used to analyze a wide range of samples, including solids, liquids, and gases, in the areas of food safety [6, 7], environmental monitoring [8, 9], fragrance analysis [10–12], forensics [13–19], pharmaceuticals [20], trace evidence analysis [21–23], and natural product research [12, 24–29]. Because it enables the rapid analysis of samples without extensive sample clean-up, DART-MS/MS is highly suitable for high-throughput screening and on-site analysis settings. However, not all compounds are compatible with DART. Chemical structure and molecular weight (approximately 100–800 Da), the presence of certain functional groups (e.g., alcohols, amines, and carboxylic acids), high volatility, low surface interaction (i.e., samples easily desorbed from glass, polymers, or plant materials), and ideal atmospheric conditions (presence of water, solvent vapor) are some of the aspects that play key roles in the ionization of compounds using the DART technique [30].

Comparing quantitative method performance characteristics, such as sensitivity and specificity, of DART-MS/MS with those of liquid chromatography–tandem mass spectrometry (LC-MS/MS) as quantitative analytical tools highlights the inherent resolving power provided by the LC column prior to MS-based detection. Many specificity issues encountered using DART-MS are addressed by identifying unique fragmentation patterns not commonly used in traditional chromatography-based methods but may provide the level of accuracy required for confident analyte identification. However, a lack of resolution between isobaric compounds, interference from matrix components or the ambient environment, and ion suppression or enhancement can limit quantitative DART-MS/MS assay development [28].

To address limitations in DART-MS/MS performance on biological matrices, particularly those arising from a lack of chromatographic separation, sample preparation can play a critical role in improving selectivity and reducing matrix effects. Beyond basic protein precipitation, several approaches have been explored. Liquid–liquid extraction (LLE) using single organic solvents, or combinations of polar and moderately polar solvents, has shown potential for enhancing analyte selectivity [18]. Solid-phase microextraction (SPME) and solid-phase extraction (SPE) [31–35] have been used to purify biological samples; in other cases, sample positioning prior to DART-MS/MS has enhanced detectability and specificity [36].

In this proof-of-concept study, we evaluated the performance of DART-MS/MS in quantitatively measuring selected antifungals [i.e., 5-fluorocytosine (5FC), itraconazole (ITR) and its metabolite hydroxyitraconazole (OH-ITR), isavuconazole (ISV), ketoconazole (KETO), fluconazole (FLU), and posaconazole (POSA)] in human serum. These drugs are among the most frequently used for treating serious fungal infections, and therapeutic drug monitoring (TDM) is recommended to help ensure efficacy while limiting toxicity [37–41]. Multiple sample preparation and analysis variables were evaluated, including protein precipitation and liquid–liquid extraction for sample cleanup and alternative ion selection for quantitative measurements. These initial optimizations were used to compare DART-MS/MS results with clinically validated LC-MS/MS results.

To our knowledge, this work is the first known application of DART-MS/MS for the quantitative determination of multiple clinically relevant antifungal agents in human serum with method comparison to LC-MS/MS.

## 2. Materials and Methods

### 2.1. Standards and Controls

Stock solutions (1 mg/mL) of 5FC (>99% purity), KETO (>98% purity) (Biomol, Hamburg, Germany), ITR, OH-ITR, FLU (>95% purity, Toronto Research Company, ON, Canada), ISV (98% purity, ACHEMBLOCK, Hayward, CA), and POSA (>98% purity, Sigma-Aldrich, Seelze, Germany) were prepared in methanol. 5FC-[^13^C_1_,^15^N_2_], ITR-[^2^H_5_], KETO-[^2^H_8_], OH-ITR-[^2^H_5_] (>95% purity, Toronto Research Chemicals, ON, Canada), ISV-[^13^C_1_, ^2^H_4_] (98% purity, Alsachim, Graffenstaden, France), POSA-[^2^H_4_], and FLU-[^2^H_4_] (99% purity, CDN Isotopes, Quebec, Canada) were used as internal standards for 5FC, ITR, KETO, OH-ITR, ISV, POSA, and FLU, respectively. A working calibration standard containing a mix of all of the analytes was prepared by spiking negative control samples (Off the Clot human serum, Golden West BioSolutions, Temecula, CA) at 5.00, 40.0, and 100 µg/mL (5FC), 0.12, 1.00, and 3.00 µg/mL (ITR), 0.20, 1.30, and 4.00 µg/mL (OH-ITR), 0.50, 3.50, and 10.0 µg/mL (ISV), 0.50, 3.00, and 9.00 µg/mL (KETO), 0.70, 5.00, and 15.0 µg/mL (FLU), and 0.25, 1.8, and 5.5 µg/mL (POSA). A working internal standard mixture was prepared in acetonitrile at 40 µg/mL (5FC-[^13^C_1_,^15^N_2_]), 3.00 µg/mL (ITR-[^2^H_5_]), 3.75 µg/mL (OH-ITR-[^2^H_5_]), 8.00 µg/mL (ISV-[^13^C_1_, ^2^H_4_] and KETO-[^2^H_8_]), 12.0 µg/mL (FLU-[^2^H_4_]), and 4.00 µg/mL (POSA-[^2^H_4_]). Quality control (QC) samples were prepared by spiking 21.1 and 50.3 µg/mL (5FC), 0.59 and 1.41 µg/mL (ITR), 0.73 and 1.72 µg/mL (OH-ITR), 1.99 and 4.59 µg/mL (ISV), 1.73 and 3.92 µg/mL (KETO), 2.7 and 6.14 µg/mL (FLU), and 0.92 and 2.25 µg/mL (POSA), respectively, in Off the Clot human serum (Golden West BioSolutions, Temecula, CA). OH-ITR-[^2^H_5_] (Toronto Research Company, ON, Canada) was also evaluated as a surrogate internal standard for POSA during secondary evaluation. All working solutions were stored at –80 °C until use. The analytical measurement ranges (AMR) evaluated for analytes were 5FC (5–100 µg/mL), ITR and OH-ITR (0.1–5 µg/mL), FLU (0.5–15 µg/mL), ISV (0.5–10 µg/mL), and POSA (0.1–5 µg/mL). The molecular structures for the analytes are shown in Figures 1a–g.

**Figure 1.**
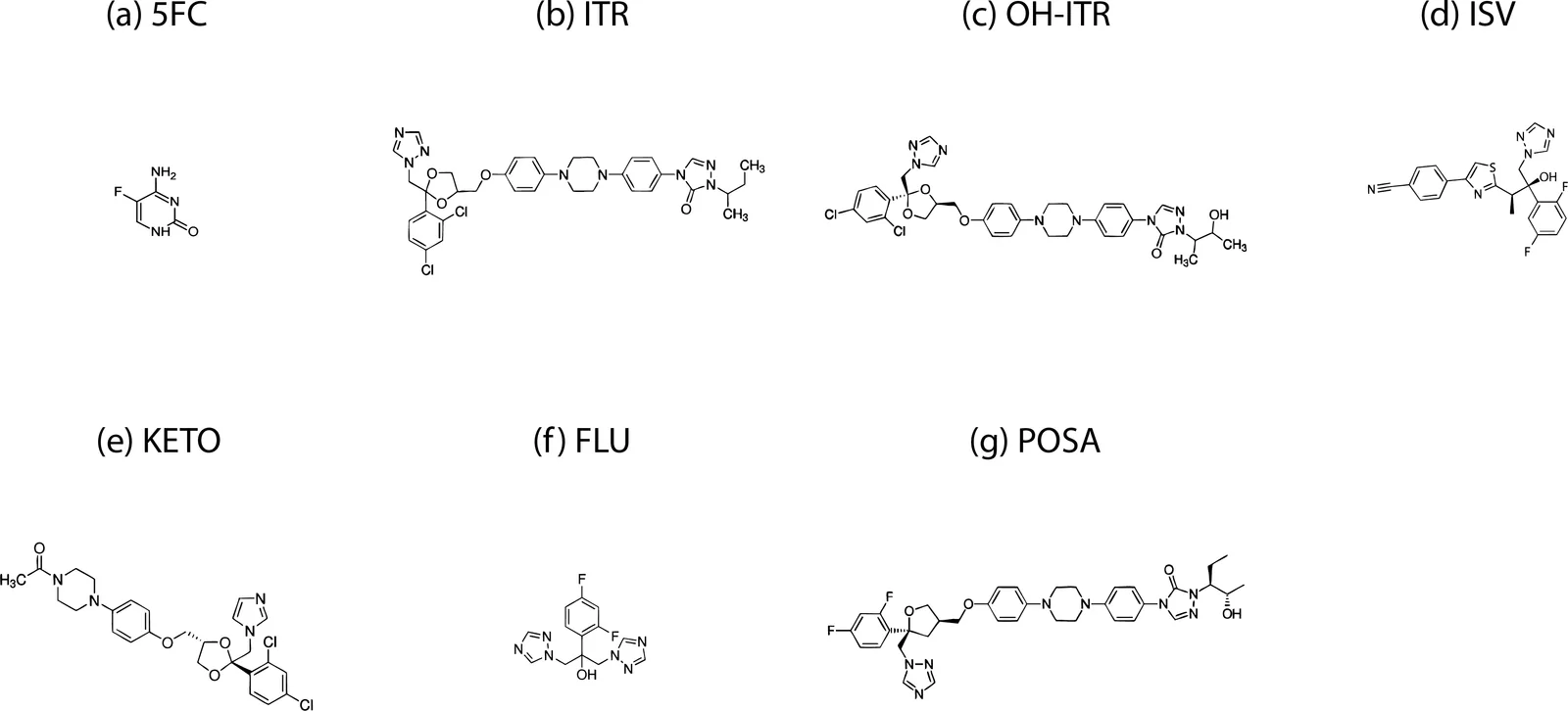
Molecular structures of analytes.

### 2.2. Sample Preparation

The use of residual samples from anonymized human donors was approved by the Institutional Review Board of the University of Utah (IRB #7275). Fifty microliters of three matrix-matched calibrators, three QC samples per analyte, and authentic patient samples were spiked with a 100 µL mixture of internal standards and subjected to protein precipitation for 5FC, ITR, OH-ITR, ISV, POSA, and KETO. Fifty microliters of matrix-matched calibrators, two controls per analyte, and patient samples were spiked with 10 µL FLU-[^2^H_4_] and subjected to LLE using 90 µL ethyl acetate for FLU. Fifty microliters of matrix-matched calibrators, two controls per analyte, and patient samples were also spiked with 50 µL OH-ITR-[^2^H_5_] (1.00 µg/mL in methanol) and subjected to LLE using 500 µL methyl-*tert*-butyl ether (MTBE) for secondary extraction experiments associated with POSA. All samples were vigorously mixed for 30 s and centrifuged at 1729 g for 5 min. The supernatant (3.5 µL, extracted by protein precipitation) was spotted onto a QuickStrip HTS 96 well DART sample plate (Bruker, Bremen, Germany) and dried at 40 °C for 20 min under nitrogen. For FLU extracted with ethyl acetate, 3.5 µL of the supernatant was directly spotted onto the DART sample plate. For POSA samples extracted with MTBE, 400 µL of the organic layer was removed, dried down under nitrogen, then reconstituted with a 50 µL mixture of 1:1 methanol: water; 3.5 µL of the reconstituted sample was then spotted onto the DART sample plate. Control samples were extracted in triplicate over 3 days. All samples were spotted in triplicate onto the DART sample plate.

### 2.3. DART-MS/MS Instrumentation and Conditions

To identify unique MS/MS transitions, the automated multiple reaction monitoring (MRM) optimization function was used to directly infuse standard solutions of analytes and associated internal standards into an EVOQ® DART-TQ+ (Bremen, Germany) triple-quadrupole MS fitted with a heated electrospray ionization (HESI) source. Five transitions per analyte were collected for use in DART-MS/MS optimization work. For DART-MS/MS analysis, the MS was fitted with an integrated DART JumpShot® HTS systems ionization source (Bremen, Germany). To identify optimal MS/MS transitions, 3.5 µL methanolic aliquots of standard solutions of analytes and associated internal standards were spotted onto QuickStrip HTS-96 Sample Cards (Bremen, Germany), dried under nitrogen for 15 min at 40 °C, and analyzed by DART-MS/MS. At least two MS/MS transitions (quantitative and qualitative) were monitored for all of the analytes and internal standards. They were initially selected on the basis of the maximum signal and minimal interferences from the ambient environment. To identify isobaric interferences between defined transitions, QuickStrip HTS-96 cards were prepared with individual analyte standards as described above, followed by DART-MS/MS analysis including all analytes in the method. The resolutions of quadrupoles Q1 and Q3 were set to 0.7. The DART ionization conditions for the fragments were optimized. The DART cone temperature and gas stream temperature were 350 °C and 375 °C, respectively, except for FLU (275 °C and 300 °C, respectively). The detector was set to dynamic mode, and the collision-induced dissociation pressure was 2.0 mtorr (0.27 Pa). The DART cone voltage and gas pressure were set to 50 V and 23 psi (159 kPa), respectively. DART was operated in linear scan mode (speed 0.5 mm/s, range 5.0 mm, as suggested by the manufacturer), and helium was used as the carrier gas. The sample acquisition time was 0.24 min. Monitored transitions and collision energies (CEs) for the analytes and respective internal standards are listed in Table 1.

**Table 1.** Monitored transitions, collision energies (CEs) for analytes, and their respective internal standards.

Analyte	Quantitative ion (CE)	Qualitative ion (CE)	Internal standard (quantitative ion, CE)
Fluconazole	306.9/238.0 (7.9)	306.9/121.0 (34.5)	FLU-[^2^H_4_] (310.9/242.1, CE 7.9)
Itraconazole	705.2/392.2 (29.6)	705.2/255.7 (29.6)	ITR-[^2^H_5_] (710.2/397.2, CE 29.6)
Hydroxyitraconazole	721.2/408.2 (34.5)	721.2/256.2 (33.6)	OH-ITR-[^2^H_5_] (726.2/413.2, CE 34.5)
Posaconazole	701.3/614.2 (26.6), 701.3/ 683.1 (24.7)	701.3/127.0 (52.3)	POSA-[^2^H_4_] (705.3/618.3, CE 30.6) OH-ITR-[^2^H_5_] (726.2/413.2, CE 34.5)
5-Fluorocytosine	130.1/113.1 (14.8)	130.1/58.1 (31.6)	5FC-[^13^C_1_, ^15^N_2_] (133.1/115.1, CE 14.8)
Ketoconazole	531.1/489.1 (26.6)	531.1/ 244.0 (35.3)	KETO-[^2^H_8_] (539.1/497.1, CE 26.6)
Isavuconazole	438.5/224.0 (16.8)	438.5/ 127 (38.5)	ISV-[^13^C_1_, ^2^H_4_] (442.1/224.0, CE 12.8)

For evaluating improved specificity, two quantitative transitions were monitored for POSA: m/z 701.3/614.2 (CE 26.6), 701.3/683.1 (CE 24.7), and a qualitative ion 701.3/127.0 (CE 52.3). POSA extractions were performed separately by protein precipitation and LLE. For the method comparison of POSA with a clinically validated LC-MS/MS method (which used OH-ITR-[^2^H_5_] as its internal standard), both OH-ITR-[^2^H_5_] and POSA-[^2^H_4_] were evaluated as internal standards. Data were acquired and analyzed using the TQControl software version 2.3.3 (Build 68).

Ion ratios were determined by dividing the peak area of the qualifier ion by that of the quantifier ion. Ion ratio tolerances, established by analyzing average ion ratios from three replicates of the calibration curve, were maintained at 50% of that observed in the matrix-matched calibration curve of each analyte (Table 2).

**Table 2.** Ion ratio tolerance of analytes.

Analyte	Ion ratio tolerance
Fluconazole	0.10–0.30
Itraconazole	0.15–0.45
Hydroxyitraconazole	0.22–0.65
Posaconazole	0.53–1.58
5-Fluorocytosine	0.18–0.53
Ketoconazole	0.23–0.68
Isavuconazole	0.15–0.44

### 2.4. Assay Performance Characteristics

Five air blanks (analysis performed with only DART gas flowing, without the introduction of any sample) were included at the beginning of each experiment. DART reproducibility was tested by spotting at least three replicates of calibrators, negative control, QC, and patient samples. Linearity and precision across the AMR were tested for all of the analytes. DART-MS/MS results for 5FC, ITR, OH-ITR, FLU, and POSA were compared with the corresponding LC-MS/MS results.

## 3. Results

The lower limit of measurement interval (LLMI) and upper limit of measurement interval (ULMI) were 5.23 and 111.0 µg/mL (5FC), 0.13 and 2.98 µg/mL (ITR), 0.18 and 3.91 µg/mL (OH-ITR), 0.45 and 10.20 µg/mL (ISV), 0.40 and 8.84 µg/mL (KETO), 0.67 and 14.1 µg/ml (FLU), and 0.23 and 5.420 µg/mL (POSA), respectively. DART chronograms/acquisitions at the LLMI of the method are shown for all analytes in Figures 2a–h. The coefficient of determination (*R*^2^) of calibration curves for all analytes (linear fit with 1/*x* weighting) is shown in supplemental Table 1. Within-run, between-run/day, and total imprecision coefficients of variation (CVs) were evaluated using two QC samples for each analyte and are reported in Table 3. Recovery of analytes spiked into matrix-matched clean negative control showed that protein precipitation as a sample preparation technique worked well for most of the analytes.

**Figure 2.**
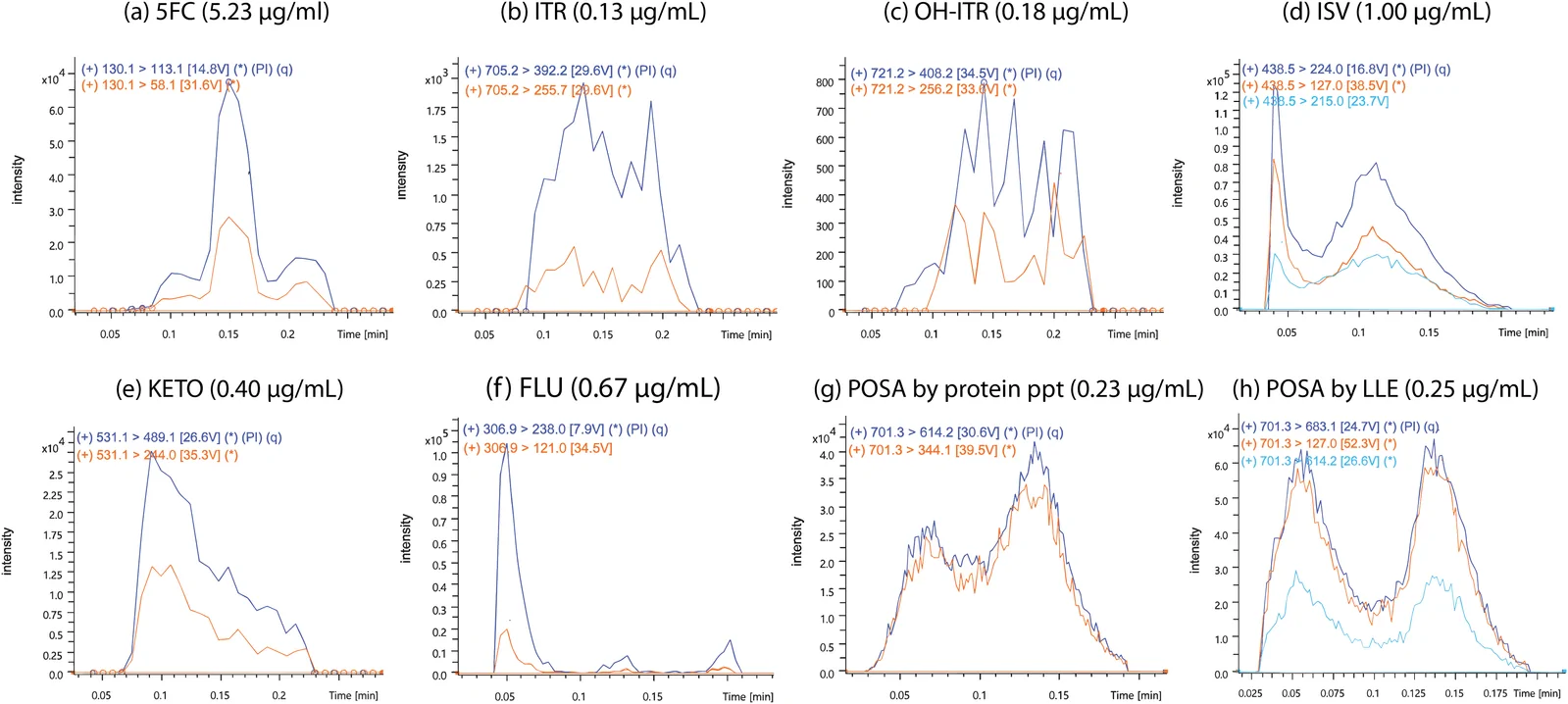
DART chronograms/acquisitions at Lower Limit of Measurement Interval (LLMI).

**Table 3.** Within-run, between-run, and total imprecision coefficients of variation (CVs) and the percent accuracy in quality control samples (QC 1 and 2).

Analyte	Within-run CV (%)	Between-run/day CV (%)	Total CV (%)	Average µg/mL	Expected µg/mL	Accuracy (%)
5FC QC 1	2.86	0.10	2.86	21.1	21.1	99.8
5FC QC 2	2.37	0.84	2.51	50.2	50.3	99.8
ITR QC 1	2.98	2.72	4.04	0.58	0.59	98.3
ITR QC 2	2.64	6.82	7.31	1.39	1.41	98.6
OH-ITR QC 1	6.14	0.83	6.19	0.73	0.73	100.1
OH-ITR QC 2	2.21	2.54	3.37	1.73	1.72	100.6
ISV QC 1	7.94	6.00	9.95	2.21	1.99	111.0
ISV QC 2	10.1	12.8	16.37	4.31	4.59	93.9
KETO QC 1	3.20	0.14	3.20	1.63	1.73	94.2
KETO QC 2	2.02	1.37	2.44	3.89	3.92	99.2
FLU QC 1	13.2	6.25	14.6	3.05	2.70	113.0
FLU QC 2	8.56	4.87	9.85	6.48	6.14	105.5
POSA QC 1	4.54	2.69	5.28	0.93	0.92	100.9
POSA QC 2	1.38	4.02	4.25	2.24	2.25	99.6

Good agreement was observed with existing LC-MS/MS methods for 5FC (slope = 1.083, *r* = 0.949, bias = 3.87%, *n* = 27), ITR (slope = 1.112, *r* = 0.975, bias = 12.9%, *n* = 37), OH-ITR (slope = 0.968, *r* = 0.950, bias = 0.8%, *n* = 37), and FLU (slope = 0.875, *r* = 0.979, bias = –3.46, *n* = 17) shown in individual scatter and Bland–Altman plots (where the *x* axis represents the mean concentration measured by LC-MS/MS and DART-MS/MS and the *y* axis represents the difference between LC-MS/MS and DART-MS/MS quantitative measurements) in Figures 3a–d, respectively.

**Figure 3.**
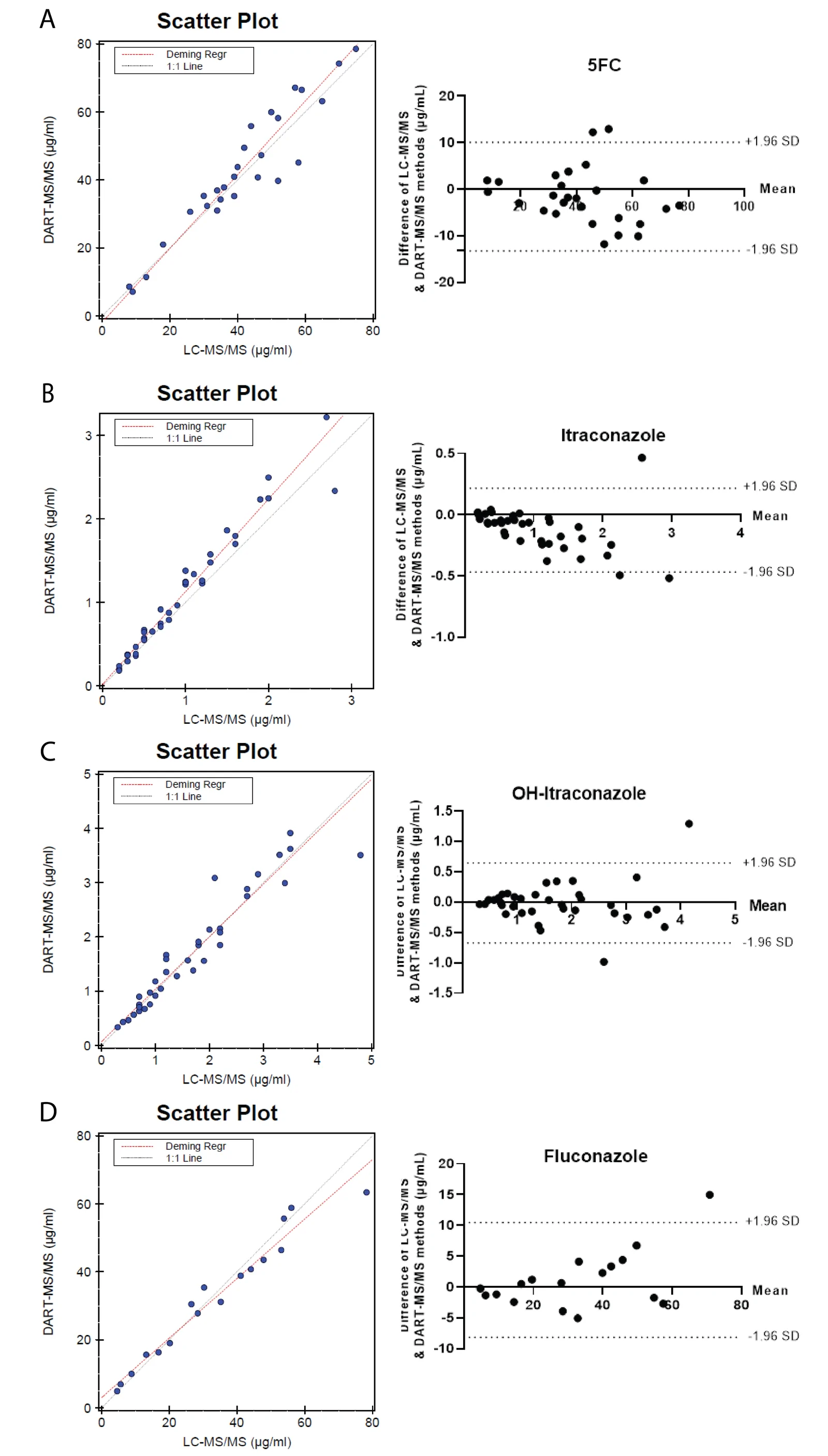
Scatter and Bland–Altman plots for DART-MS/MS vs comparative LC-MS/MS method: a) 5FC (slope = 1.083, *r* = 0.949, bias = 3.87%, *n* = 27), b) ITR (slope = 1.112, *r* = 0.975, bias = 12.9%, *n* = 37), c) OH-ITR (slope = 0.968, *r* = 0.950, bias = 0.80%, *n* = 37), d) FLU (slope = 0.875, *r *= 0.979, bias = −3.46, *n* = 17).

The DART-MS/MS results for POSA samples extracted by protein precipitation showed a high positive bias when compared with the results obtained using an existing LC-MS/MS method, although the relationship was proportional (slope = 2.302, *r* = 0.845, bias = 79.9%, *n* = 38), as shown in scatter and Bland–Altman plots (Figure 4a). When LLE was used with MTBE, the POSA transition 701.3/614.2 showed slope = 0.930, *r* = 0.993, bias = –0.6%, *n* = 30 (Figure 4b); when a second transition, 701.3/683.1, was used, method comparison with LC-MS/MS results showed slope = 0.896, *r* = 0.957, bias = 6.76%, *n* = 29 (Figure 4c). Supplemental Figure 1 shows both the 614.2 and 683.1 fragments obtained from the ionization of POSA. Although the average percent accuracy in the LLE results when 701.3/614.2 was used as the quant ion was 102% compared with that obtained by LC-MS/MS, 3 out of 30 samples showed >30% bias when DART-MS/MS was used, even though the ion ratios in the 3 samples remained within acceptable tolerance (0.53–1.58).

**Figure 4.**
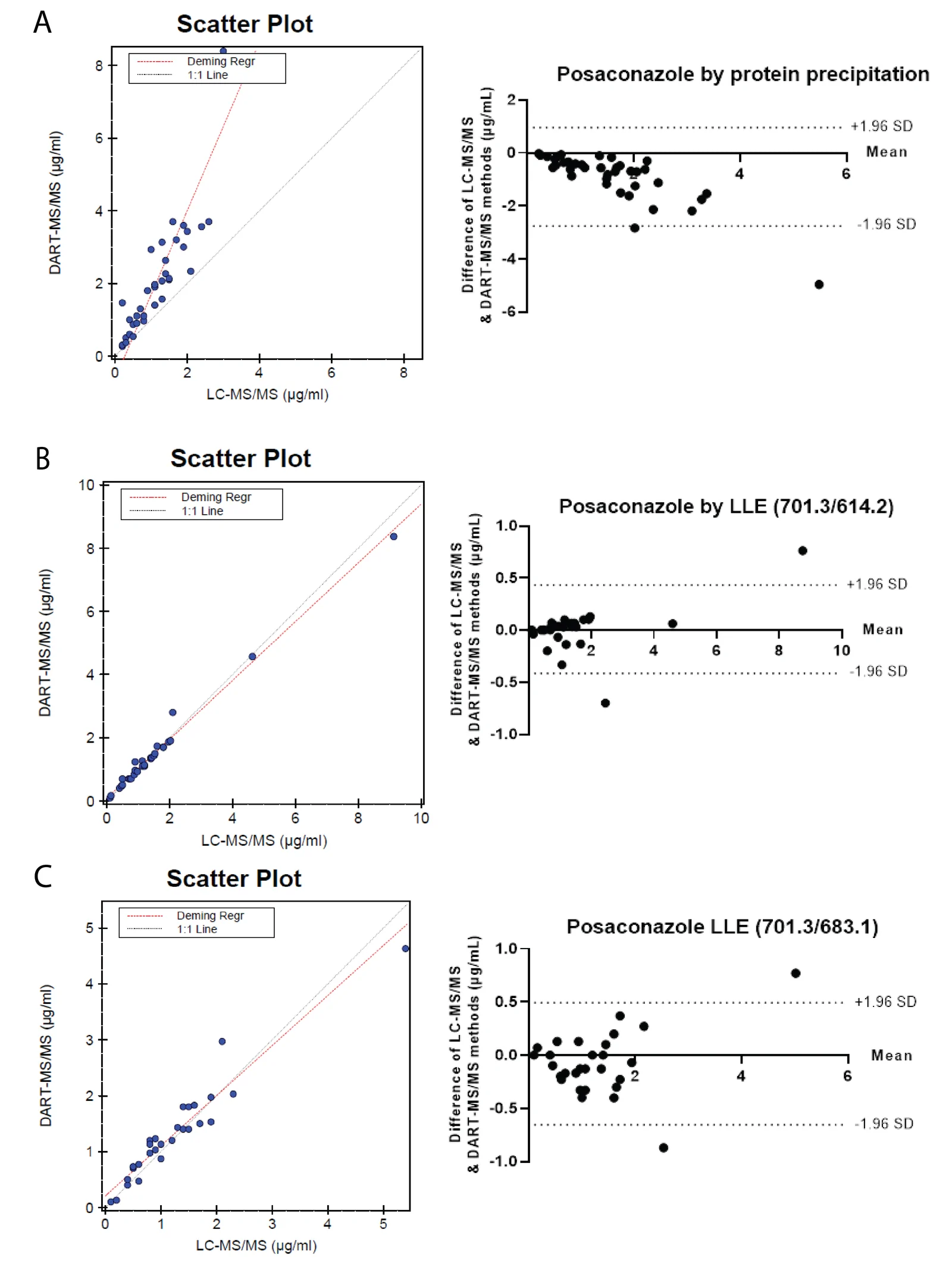
Scatter and Bland–Altman plots for DART-MS/MS vs comparative LC-MS/MS method: a) POSA by protein precipitation and quant ion 701.3/614.2 (slope = 2.302, *r* = 0.845, bias = 79.9%, *n* = 38), b) POSA by LLE and quant ion 701.3/614.2 (slope = 0.930, *r* = 0.993, bias = −0.64%, *n* = 30), c) POSA by LLE, transition 701.3/683.1 (slope = 0.896, *r* = 0.957, bias = 6.76%, *n* = 29).

The imprecision (CV) of DART replicates was found to be <15% for all of the samples and analytes. A higher CV was observed in some spots where the manual spotting did not land in the center of the sample positions in the DART sample plate. To account for this variability, we carried out DART in linear scan mode to ensure that sufficient sample was analyzed.

No carryover was observed between negative controls placed immediately after the highest calibrator sample. The internal standard contribution at LLOQ was <20% for all of the analytes. All of the samples passed the ion-ratio tolerance maintained at ±50%.

The difference in time required for analyzing each sample by DART-MS/MS versus the clinically validated comparative LC-MS/MS method is noteworthy. The acquisition time per sample was 0.24 min by DART-MS/MS, whereas it was 2.5 min by the comparative LC-MS/MS method. Ninety-six individual samples could be analyzed in approximately 23 min vs 4 h by LC-MS/MS.

Finally, although laboratories consider moving from helium to nitrogen as the source gas for DART to mitigate expenses, we did not evaluate nitrogen because, early in our work on these analytes, we noticed that analyte peak areas were 10-fold lower when nitrogen was used instead of helium.

## 4. Discussion

Herein, a fast and simple DART-MS/MS method for the quantitative measurement of antifungals 5FC, ITR, OH-ITR, ISV, KETO, FLU, and POSA in human serum was developed and evaluated. The molecular weights of these compounds range from 129.09 (5FC) to 721.6 g/mol (OH-ITR). Structurally, 5FC is a pyrimidine analogue, whereas ITR, OH-ITR, ISV, KETO, FLU, and POSA are triazoles; both groups exhibit basic properties in the gas phase, making them well suited for DART ionization in positive mode. All of the assessed compounds were readily ionizable under the DART-MS conditions. The method could detect and quantify the analytes found in biological samples and showed ample sensitivity for measurement in clinically relevant concentrations in human serum.

The combination of chromatographic separation and tandem mass spectrometry makes LC-MS/MS a highly selective and sensitive analytical tool for the identification and quantification of target analytes in complex matrices. It is a widely established technique with well-developed protocols and readily available expertise. When used in conjunction with internal standards, LC-MS/MS analysis often leads to accurate and precise quantitation of multiple analytes through monitoring of ion ratios and evaluation of retention times, with minimal sample preparation. When LC-MS analysis is difficult because of issues such as poorly ionizing compounds or extensive sample cleanup requirements, DART-MS/MS offers a strong alternative. It provides real-time analysis under ambient conditions, with versatile sample handling for solids, liquids, and gases on different surfaces. Its minimal sample consumption, reduced solvent use, and broad applicability make DART-MS/MS especially suitable for situations where quick results are critical.

In this study of quantification of antifungals by DART-MS/MS, we mostly evaluated protein precipitation as a rapid sample preparation method (5FC, ITR, OH-ITR, ISV, KETO); however, in some cases (FLU and POSA), LLE was found to improve performance. In addition, to confirm the initial success of our proof-of-concept study, we ensured that each analyte was associated with its own stable isotopically labeled internal standard, thus warranting measurement accuracy in a quantitative application. Comparison of the DART-MS/MS method with validated LC-MS/MS methods (based on protein precipitation using an organic solvent, while using reverse-phase chromatography with the MS system operated in positive mode), demonstrated reasonable correlation for 5FC, ITR, OH-ITR (using protein precipitation), and FLU and POSA (by LLE). Validated LC-MS/MS methods to compare DART-MS/MS results for KETO and ISV were not available for the present study.

For the POSA method comparison experiment, we began with POSA-[^2^H_4_] as the internal standard and compared the results with those obtained using a clinically validated LC-MS/MS method (which used OH-ITR-[^2^H_5_] as an internal standard). Thirty-eight individual patient samples, with positive results for POSA by LC-MS/MS, extracted by protein precipitation showed very high bias (average of 79.9% higher by DART-MS/MS). Assuming that the surrogate internal standard in the compared LC-MS/MS method could be the cause of the high bias, we replaced POSA-[^2^H_4_] with OH-ITR-[^2^H_5_] as the internal standard to mimic the comparative LC-MS/MS method. This change did not alleviate the observed bias, which led us to hypothesize that, in the case of POSA, DART-MS/MS was unable to resolve interferences from structurally similar antifungals, anticoagulants, antibiotics (e.g., cefepime, piperacillin, ceftazidime, trimethoprim, and rifampin [42, 43]), or other endogenous compounds.

To further evaluate the specificity of POSA detection, we changed the sample preparation technique from protein precipitation to LLE. POSA has a Log*P* value of 4.6 and poor solubility in water even though it exhibits both polar and non-polar characteristics. We considered exploiting this nature of the analyte by extracting the samples using MTBE. LLE necessitates drying the organic layer and reconstituting the sample in a solvent prior to its deposition, often leading to sample loss. However, because the CVs between replicate peak areas of POSA chronograms/acquisitions at the LLMI using LLE were ample (Figure 2g), we extracted the samples using MTBE, dried them, and then reconstituted them in 1:1 water methanol. For a higher level of specificity, we evaluated two quantitative ions for POSA (701.3/ 614.2 and 701.3/683.1). The use of either of the quantitative ions combined with LLE provided remarkable improvement in specificity of POSA by reducing the bias from 79.9% (by protein precipitation) to –0.64% (using quant ion 701.3/614.2) and 6.76% (using quant ion 701.3/683.1) by LLE. These results proved our hypothesis that sample cleanup could substantially improve specificity and performance in DART-MS/MS. We also noticed that when we used LLE and 701.3/614.2 as the primary ion, 3 samples (10%) among the 30 patient samples used for comparison showed accuracies ranging from 142% to 150%. The concentration of these samples ranged from 0.5 to 2.1 µg/mL (LLMI 0.23 µg/mL). The ion ratios of these samples did not show any failures (remaining within the acceptable range for POSA). Thirty percent of samples failed accuracy by LLE when 701.3/683.1 was used as the primary ion, which led us to conclude that the samples could contain additional interferences not resolved by LLE.

Another consideration for improving the specificity in DART measurements is the precision of sample deposition onto the DART sample plate and, thereafter, the DART sampling mode used in the method. Given that we were manually depositing small sample volumes (2–3.5 µL), the sample spots were not likely to have been exactly centered. In relation to sample deposition and sampling, we observed that, in our hands, replicates of peak areas of chronograms/acquisitions and CVs were consistently better (<15%) when the DART linear scan mode was used instead of the pulsed JumpShot mode (circular scan mode was not available for use but could be evaluated in future work). The linear scan mode permits a longer interaction time of the sample with ambient background molecules, thereby increasing the chance of interference when compared with a pulsed scan mode. Automated sample deposition and JumpShot scanning could be further evaluated to test observed POSA inaccuracies. In some reported cases, adjusting and optimizing DART cone voltages or using a lower DART temperature method before using a high-temperature method has helped remove some commonly found low-boiling-point interferences [44]. Solid-phase microextraction (SPME), solid-phase extraction (SPE), high-temperature thermal desorption [44], sample dilution, ion mobility spectrometry [45], and high-resolution mass spectrometers [46] have improved DART performance for some analytes, suggesting that these could be evaluated for further optimization of POSA testing. The >15% total CV observed in the case of high ISV QC 2 (16.37%) can also be attributed to incorrect sample deposition leading to a difference in ionization efficiency and, therefore, to different signal intensities between days of measurement. Automation of the sample deposition process could be evaluated to reduce this variability [47].

LLE-extracted POSA samples reconstituted in 1:1 water:methanol showed less variation between DART replicates compared with those reconstituted in methanol only. This difference is attributable to a lower evaporation rate when using a mixture of methanol and water versus samples dissolved solely in methanol.

The smaller analyte peak areas associated with nitrogen instead of helium being used as the DART gas are attributable to a lower thermal conductivity and lower flow velocity associated with diatomic nitrogen (higher density). Helium’s higher thermal conductivity, by contrast, increased both the ionization efficiency and the sensitivity, making it a more suitable DART gas during this evaluation [48].

This study demonstrates that enhanced sample preparation protocols in conjunction with DART-MS/MS can support its broader applicability to diverse drug classes and reinforce its value as a rapid, high-throughput analytical tool.

## 5. Conclusion

We developed and tested assay performance characteristics for a DART-MS/MS method for measuring the antifungals 5FC, ITR, OH-ITR, ISV, KETO, FLU, and POSA in human serum. We compared the results with those of validated LC-MS/MS methods. The improvement in specificity afforded by sample cleanup and/or the use of a different fragment ion shows that DART-MS/MS holds promise as a simple and fast quantitative measurement technique. This work represents the first known application of DART-MS/MS in measuring multiple antifungals in human serum with method comparison to LC-MS/MS.

## Data Availability

The data underlying the study are available from the corresponding author upon reasonable request.

## Abbreviations

5FC: flucytosine
AMR: analytical measurement range
AP-MALDI: atmospheric-pressure MALDI
CE: collision energy
CID: collision-induced dissociation
CV: coefficient of variation
DART: direct analysis in real time
DART-MS: direct analysis in real time mass spectrometry
DART-MS/MS: direct analysis in real time tandem mass spectrometry
Da: dalton
DESI: desorption electrospray ionization
FLU: fluconazole
HESI: heated electrospray ionization
IMS: ion mobility spectrometry
IRB: institutional review board
ISV: isavuconazole
ITR: itraconazole
KETO: ketoconazole
LC-MS: liquid chromatography–mass spectrometry
LC-MS/MS: liquid chromatography–tandem mass spectrometry
LLE: liquid–liquid extraction
LLMI: lower limit of measurement interval
LLOQ: lower limit of quantification
MALDI: matrix-assisted laser desorption/ionization
MRM: multiple reaction monitoring
MS: mass spectrometry
MS/MS: tandem mass spectrometry
MTBE: methyl *tert*-butyl ether
OH-ITR: hydroxyitraconazole
POSA: posaconazole
QC: quality control
QTOF-MS: quadrupole time-of-flight mass spectrometry
RUO: research use only
SPE: solid-phase extraction
SPME: solid-phase microextraction
SPMESH: solid-phase microextraction sheets
TDM: therapeutic drug monitoring
ULMI: upper limit of measurement interval
